# Optimizing diabetes self-care in patients with limited health literacy: a randomized controlled trial (RCT) of SCT-based education with and without an AI-designed photo-novel

**DOI:** 10.1186/s12913-026-14849-9

**Published:** 2026-06-05

**Authors:** Tooba Chekav, Homamodin Javadzade, Marzieh Mahmoodi, Mahnoush Reisi

**Affiliations:** 1 Department of Health Education and Health Promotion, Bushehr University of Medical Sciences, Bushehr, Iran; 2 Addiction and Lifestyle Research Center, Bushehr University of Medical Sciences, Bushehr, Iran; 3 Department of Biostatistics and Epidemiology, Bushehr University of Medical Sciences, Bushehr, Iran

**Keywords:** Social cognitive theory, Type 2 diabetes, Self-care, Photo novel, Health literacy

## Abstract

**Background:**

Type 2 diabetes mellitus (T2DM) requires continuous self-care to prevent complications. Patients with limited health literacy often struggle to understand health information and adhere to treatment, resulting in poorer outcomes. Educational interventions tailored to improve self-care and health literacy arSCTe therefore essential. This study evaluated the effectiveness of a Social Cognitive Theory-based intervention, with and without health literacy strategies, in enhancing self-care among patients with T2DM and limited health literacy.

**Methods:**

This interventional study included 150 patients with T2DM and limited health literacy, randomly assigned to three equal groups (*n* = 50 each): a Social Cognitive Theory-based group (TBG), a theory-based group with an AI-designed photo-novel (TBG+AIPN), and a control group. At baseline, all participants completed questionnaires assessing diabetes knowledge, self-efficacy, outcome expectations, perceived social support, and self-care behaviors. Both intervention groups received five training sessions grounded in Social Cognitive Theory. In the TBG+AIPN group, additional health literacy strategies—such as an AI-designed photo-novel and plain language materials—were incorporated. Follow-up assessments were conducted one and three months post-intervention. Data were analyzed using SPSS version 24.

**Results:**

Longitudinal analyses revealed significant within-group improvements from baseline to follow-up across all measured constructs in both intervention groups (all *p* < 0.001). Between-group comparisons showed that the TBG+AIPN group achieved significantly greater improvements than the TBG group in self-care behaviors (*p* = 0.002), diabetes knowledge (*p* = 0.040), self-efficacy (*p* = 0.020), outcome expectations (*p* = 0.030), and self-regulation (*p* = 0.020). No statistically significant difference was observed between the groups in perceived social support (*p* = 0.800). These findings highlight the added value of integrating AI-designed visual literacy tools with SCT-based education in improving self-care–related outcomes among patients with T2DM.

**Conclusion:**

An intervention based on Social Cognitive Theory, when combined with health literacy strategies including an AI-designed photo-novel, is more effective in improving self-care among patients with T2DM and limited health literacy. Such a comprehensive approach integrates psychological and communication strategies, offering a holistic solution for improving outcomes in this vulnerable group.

**Trial registration:**

Iranian Registry of Clinical Trials (IRCT), IRCT20240426061579N1. Registered on 12 May 2024.

## Background

Diabetes is a major global health challenge affecting individuals, families, and nations alike. According to the 2025 International Diabetes Federation (IDF) Diabetes Atlas, 11.1% of adults aged 20–79—approximately one in nine people—currently live with diabetes. Projections indicate that by 2050, 1 in 8 adults, or around 853 million people, will be living with the condition, representing a 46% increase [1]. In Iran, the 2024 IDF report reveals a 9% prevalence of diabetes among adults, equating to roughly 5.4 million individuals affected by the disease [2].

Type 2 diabetes mellitus (T2DM), the predominant form of diabetes, substantially contributes to disability, premature mortality, and escalating healthcare costs. It is associated with a range of serious complications, including cardiovascular disease, stroke, retinopathy, neuropathy, nephropathy, diabetic foot ulcers, and depression [3, 4].

Although no cure currently exists, diabetes can be effectively managed through lifestyle modifications combined with medical interventions. The primary objective of diabetes management is to maintain blood glucose levels within the recommended target range in order to prevent or delay complications [5]. Management of patients with T2DM often involves dietary changes, physical activity, and medication. It is also important to regularly monitor blood sugar levels and inspect feet daily to avoid complications such as diabetic neuropathy and diabetic foot ulcers, which can lead to foot amputation if not treated in time [6].

Despite the crucial importance of blood sugar control, many patients worldwide still haven’t achieved optimal blood glucose management [7, 8]. A systematic review conducted in Iran in 2020 revealed that, on average, patients with diabetes achieved only 50% of the recommended self-care behaviors [9]. In response, the American Diabetes Association (ADA) emphasizes patient education as vital for improving self-care and reducing complications [10]. However, while education is a fundamental component, addressing the psychosocial factors of health-related behaviors is equally important for designing effective and sustainable interventions [11]. In this regard, the application of behavioral theories provides a valuable framework for identifying the underlying factors that influence health-related behaviors and for improving the efficacy of intervention strategies [12].

Social Cognitive Theory (SCT) is recognized as one of the most influential frameworks for understanding and promoting health behavior change. It has been widely applied in health science research and has demonstrated effectiveness in the development of various health intervention programs [13, 14]. SCT emphasizes the dynamic and reciprocal interaction among personal, environmental, and behavioral factors, offering a comprehensive model for explaining and predicting health behaviors [15]. Key constructs of the theory—such as self-efficacy, self-regulation, outcome expectations, and outcome valuation—play a pivotal role in influencing behavioral adoption and maintenance [14].

Although theory-based education has proven effective in enhancing self-care among patients with type 2 diabetes, considerable challenges persist—particularly for individuals with limited health literacy [16]. Health literacy, defined as the ability to read, comprehend, evaluate, and apply health-related information, plays a critical role in the success of educational interventions [17]. Evidence suggests that even after repeated exposure to standard educational content, many individuals with low health literacy remain disengaged and lack the motivation and understanding needed for effective self-management. This highlights the limitations of conventional education methods in meeting the specific needs of this vulnerable population [18].

To effectively address the challenges faced by individuals with limited health literacy, several strategies have been recommended by authoritative organizations such as the American Medical Association [19]. These include simplifying educational content, incorporating visual media, and developing health messages that are clear and easy to understand [20]. Such approaches have been shown to improve comprehension and reduce common misunderstandings among low health literacy populations [21]. On the other hand recently, the integration of artificial intelligence (AI) into healthcare has opened new avenues for enhancing patient education, particularly among vulnerable groups. AI provides powerful tools to tailor educational content to individual needs, enhancing understanding, engagement, and retention [22]. Emerging evidence shows that AI-supported patient education improves clinical outcomes, increases efficiency, and promotes equitable access to personalized care [23]. In this study, we employed an innovative approach that combined the principles of health education with AI-generated visual media in the form of a photo-novel designed specifically for individuals with limited health literacy.

Although recommended strategies for developing educational materials tailored to individuals with limited health literacy—such as simplifying content and adapting messages to patients’ comprehension levels—appear promising, evidence regarding their actual effectiveness remains limited. Specifically, there is a lack of clarity about the extent to which the use of such strategies influences the outcomes of educational interventions among patients with type 2 diabetes and limited health literacy. To address this gap, the present study aimed to compare the effectiveness of a Social Cognitive Theory-based intervention delivered with and without an AI-generated photo-novel on self-care behaviors in patients with type 2 diabetes and limited health literacy.

## Methods

### Study design

This study follows the CONSORT guidelines and was a three-arm, parallel-group randomized controlled trial (RCT) designed to evaluate the effectiveness of Social Cognitive Theory (SCT)-based educational interventions, with and without an AI-designed photo-novel, on self-care behaviors among patients with type 2 diabetes mellitus (T2DM) and limited health literacy.

### Setting and participants

The study was conducted at comprehensive health service centers affiliated with Bushehr University of Medical Sciences, Bushehr province, Iran, between April and June 2024. Eligible participants were adults aged ≥ 30 years with a confirmed diagnosis of type 2 diabetes mellitus (T2DM) for at least one year, verified through electronic medical records and based on the diagnostic criteria of the American Diabetes Association (ADA, 2023). Participants were also required to have limited health literacy, as assessed by the Short Test of Functional Health Literacy in Adults (S-TOFHLA), and the ability to read and write in Persian.

Exclusion criteria included severe diabetes-related complications (such as renal failure, vision-threatening retinopathy, or serious foot ulcers), participation in diabetes self-care education programs within the past six months, or missing more than one educational session.

### Sample size

Based on previous studies and power analysis using G*Power 3.1, a minimum of 39 participants per group was required to achieve 80% power at a significance level of α = 0.05. To account for potential attrition, 50 participants were recruited per group (total *n* = 150).

### Randomization and allocation

A cluster randomization design was used, with six comprehensive health service centers as the units of randomization. Centers were first randomly selected, after which eligible participants within each center were randomly assigned to one of three study groups:

1. SCT-based intervention group (TBG).
2. SCT-based intervention with AI-designed photo-novel (TBG + AIPN).
3. Control group receiving usual care.

**Fig. 1:**
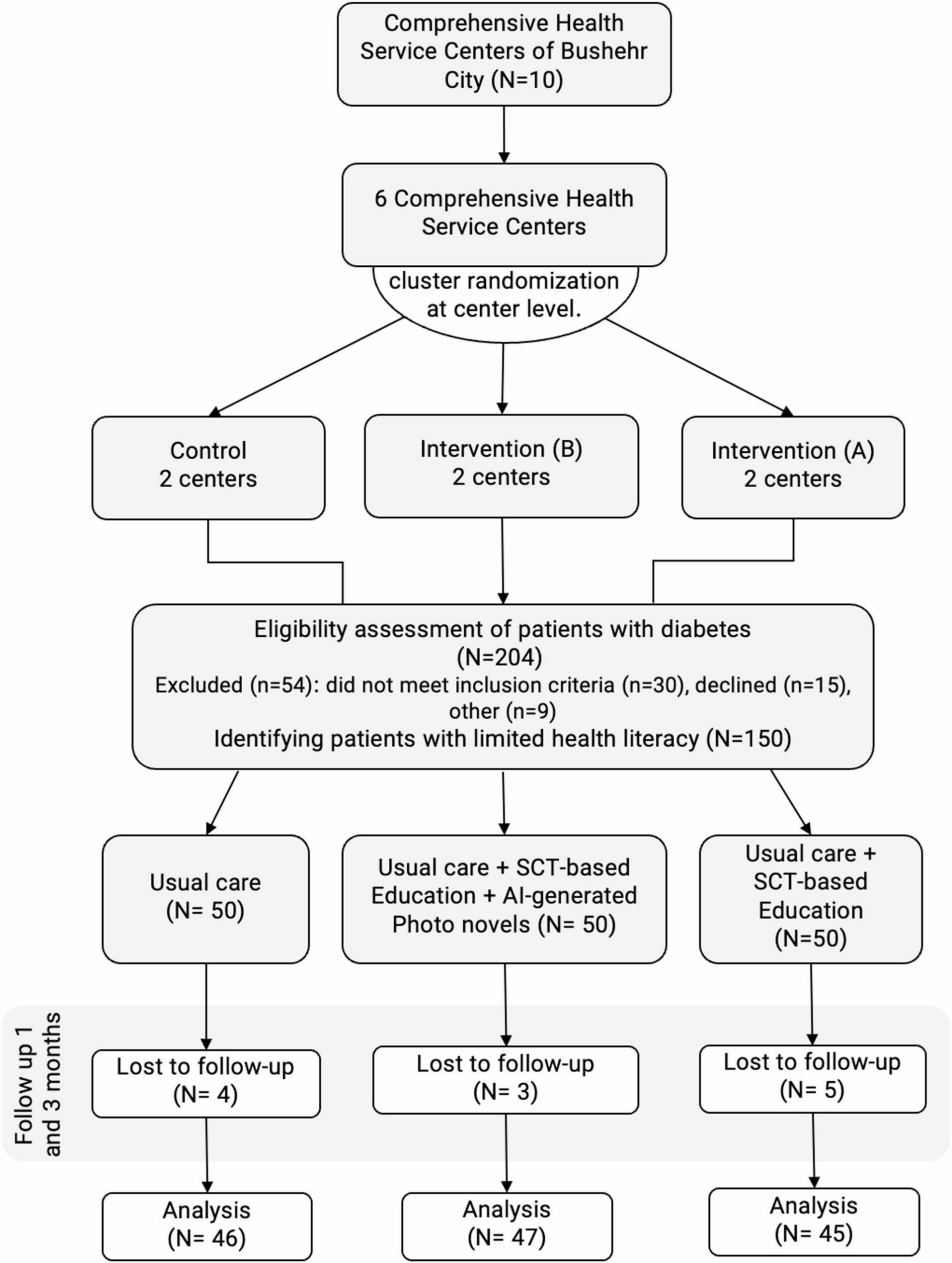
CONSORT flow diagram of participant recruitment, allocation, and follow-up

Randomization sequences were generated using R software, with allocation concealed until intervention initiation. Due to the limited number of clusters, reliable estimation of between-cluster variance was not feasible. Therefore, we performed repeated-measures analyses at the individual level, accounting for within-subject correlations across time points, consistent with methodological recommendations for studies with very few clusters. The CONSORT flow diagram is presented in Fig. 1.

### Intervention

This randomized controlled trial consisted of two intervention groups and one control group. Both intervention groups received structured training based on SCT, and the second group was additionally supported by an educational visual novel designed by artificial intelligence to accommodate participants with limited health literacy. The first intervention group (TBG) participated in five 60-minute sessions held twice a week, focusing on SCT constructs including self-efficacy, self-regulation, outcome expectations, and perceived social support. The sessions were delivered by trained facilitators using lectures, discussions, and practical activities to enhance diabetes self-management. The first session introduced type 2 diabetes, symptoms, complications, and the importance of self-care, and highlighted the consequences of nonadherence and the benefits of ongoing self-management. The second session focused on adherence to a healthy diet and medication, using step-by-step guidance and peer role models to enhance self-efficacy. The third session addressed physical activity, blood glucose monitoring, and foot care using practical demonstrations and step-by-step practice. The fourth session targeted self-regulation by reviewing previous behaviors, identifying barriers, and setting practical goals through self-monitoring and reflection. The fifth session involved family members or caregivers to reinforce perceived social support, with strategies to maintain and reinforce self-care behaviors, while facilitators used verbal encouragement to motivate participants and reinforce positive behaviors.

The second intervention group (TBG + AIPN) received the same core curriculum and TBG session structure, but included the addition of an educational visual novel designed by artificial intelligence to further support participants with limited health literacy. The visual novel was specifically developed using culturally relevant characters and realistic scenarios to illustrate self-care behaviors and decision-making in daily life. During the intervention sessions, the visual novel was used interactively, allowing participants to follow the characters’ stories, discuss their decisions, and reflect on how the concepts applied to self-care. This approach aimed to increase engagement, model positive behaviors, and provide a tangible and understandable learning experience that complemented the theoretical content of the SCT-based training. Table 1 summarizes the key components of the educational sessions, including the main objectives, core activities, and duration for each group.

The control group received standard clinical care without additional structured sessions or educational materials. All outcomes, including diabetes self-care behaviors and SCT constructs (knowledge, self-efficacy, outcome expectations, self-regulation, and perceived social support), were assessed one and three months after the intervention using standardized questionnaires. This assessment allowed for the measurement of immediate and short-term persistence of self-care behaviors and related psychological constructs. Intervention Delivery and Fidelity.

All educational sessions were conducted according to a standardized protocol. Sessions were delivered by the principal investigator, while adherence to the program by participants was monitored by the research team to ensure consistency and fidelity.

### Developing photo novel

The educational photo-novel used in this study was developed with the aid of artificial intelligence tools to enhance engagement and accessibility specifically for participants with limited health literacy. Detailed character profiles were generated using ChatGPT, including a central character with type 2 diabetes, their family members, and healthcare providers, all designed to reflect culturally relevant and relatable identities.

Initial visual representations were created using the AI platform Perchance.org. Facial expressions and emotional states were refined using Remaker.ai and FaceApp to improve realism and convey affective nuance. The final images were composed into coherent story scenes using Adobe Photoshop and Photo Leap AI.

To optimize comprehension among individuals with low health literacy, all textual content was simplified according to the Gunning Fog Index (target GFI < 6). The resulting photo-novel was distributed at the start of each educational session and served interactively as a discussion tool to illustrate and reinforce key self-care concepts through scenario-based storytelling.

The photo-novel depicted everyday challenges faced by the main character and demonstrated appropriate self-management behaviors through interactions with family members and healthcare providers, aiming to promote behavior change by increasing emotional resonance and personal relevance.

Usability and appropriateness were assessed during a pilot phase with ten patients with type 2 diabetes. Feedback from this pilot was incorporated into the final version before implementation in the intervention sessions, ensuring the tool was clear, engaging, and culturally relevant.

**Table 1:** Overview of intervention content, structure, and delivery methods across study groups

Group	Session Number	Duration	Main Content	Delivery Method	SCT Construct	Tools/ Materials
TBG	1	60 min	Introduction to diabetes, complications, and importance of self-care	Lecture and group discussion	Outcome expectations	–
TBG	2	60 min	Diet and medication adherence	Practical exercises and modeling	Self-efficacy	–
TBG	3	60 min	Physical activity, blood glucose monitoring, foot care	Practical exercises and group discussion	Self-efficacy, Outcome expectations	–
TBG	4	60 min	Self-regulation skills, goal setting, and self-assessment	Practical exercises	Self-regulation	–
TBG	5	60 min	Family involvement and social support	Group discussion and practical exercises	Perceived social support	–
TBG+AIPN	1–5	60 min per session	Same content as TBG + AI-designed educational photo-novel	Same methods + use of AI-designed photo-novel	All constructs	AI-designed photo-novel

### Instruments and measures

#### Test of Functional Health Literacy in Adults-shortened version (S-TOFHLA)

Participants’ health literacy was assessed using the abbreviated Test of Functional Health Literacy in Adults (S-TOFHLA) [24]. This tool comprises two reading passages (36 items) and four numeracy items (7 items), designed to evaluate the ability to read and understand health-related forms and labeled prescriptions. Each correct answer in both sections is awarded one point. In the reading section, each item is multiplied by 2 (36 items), resulting in a score ranging from 0 to 72, and in the numeracy section, each item is multiplied by 7 (4 items), yielding a score between 0 and 28. The total S-TOFHLA score is the sum of these two sections, ranging from 0 to 100. Scores are categorized as inadequate health literacy (0–53), marginal health literacy (54–66), and adequate health literacy (67–100). Scores on the S-TOFHLA range from 0 to 100. According to the established cut-off points for this questionnaire, scores between 0 and 66 indicate limited health literacy. The S-TOFHLA has been validated in English and other languages [25, 26] and the Persian version of the scale demonstrates adequate internal reliability, with a Cronbach’s α of 0.69 for numeracy and 0.78 for reading comprehension [27].

#### Summary of Diabetes Self-Care Activities (SDSCA)

Diabetes-related self-care behaviors were evaluated using the Summary of Diabetes Self-Care Activities (SDSCA) questionnaire [28]. This 12-item instrument captures the frequency of self-care behaviors over the preceding seven days, with each item scored from 0 (“none of the days”) to 7 (“every day”), for a total possible score of 0–84. The validity and reliability of the SDSCA have been established in previous studies [29].

#### The Diabetes Management Self-Efficacy Scale (DMSES)

Participants’ confidence in undertaking diabetes self-management tasks was assessed using the Diabetes Management Self-Efficacy Scale (DMSES) [30]. The DMSES consists of 20 items rated on a 5-point Likert scale from 1 (“I definitely can’t”) to 5 (“I definitely can”), yielding total scores ranging from 20 to 100. Higher scores reflect greater self-efficacy. The Persian version exhibits excellent reliability (Cronbach’s α = 0.92) [31].

#### Self-regulation and social support scale

To evaluate Social Cognitive Theory constructs of self-regulation and perceived social support, we administered a 27-item scale developed by the research team and adapted from Fazli et al. [32]. The instrument comprises 27 items—16 measuring self-regulation (scores ranging from 16 to 80) and 11 measuring perceived social support (scores ranging from 11 to 55)—each rated on a 5-point Likert scale (1 = “strongly disagree” to 5 = “strongly agree”). Respondents reported the frequency of each self-regulation behavior over the preceding month using a five-point scale: “never” [1], “rarely” [2], “sometimes” [3], “most of the time” [4], or “always” [5]. Content validity was established by expert review, yielding a CVR of 0.81 and a CVI of 0.90 for the perceived social support scale, and a CVR and CVI of 0.80 for the self-regulation scale. Internal consistency was acceptable, with Cronbach’s α coefficients of 0.70 for perceived social support and 0.72 for self-regulation [29].

#### Diabetes knowledge

Diabetes knowledge was evaluated using the 10-item multiple-choice questionnaire developed by Eigenmann et al. (2011) [33]. The questionnaire consists of 10 multiple-choice questions, each with four response options. Correct answers are awarded 1 point and incorrect answers are awarded 0 points. The Persian version of this questionnaire has strong psychometric properties, with consistent validity (CVI = 0.93) and reliability (Cronbach’s α = 0.73).

#### Outcome expectations

A seven-item questionnaire, adapted from Talbot et al. [34], was used to quantify participants’ anticipated benefits of diabetes self-care behaviors. Respondents indicated their level of agreement with each statement on a five-point Likert scale (1 = “strongly disagree” to 5 = “strongly agree”). The Persian version demonstrated strong content validity (CVI = 0.81) and acceptable internal consistency (Cronbach’s α = 0.75).

### Blinding

Blinding was not possible due to the nature of the intervention. Both participants and the principal investigator were aware of group assignments.

### Outcomes

#### Primary outcome

Diabetes self-care behaviors measured using a standardized questionnaire.

#### Secondary outcomes

Diabetes knowledge, self-efficacy, outcome expectations, perceived social support, and self-regulation.

Assessments were conducted at baseline, one month, and three months post-intervention.

### Data analysis

Data were coded and analyzed using R software version 4.2.2. Descriptive statistics were used to summarize the baseline characteristics of participants across the study groups. Differences in demographic variables among groups were assessed using Chi-square tests, while mean scores of SCT constructs and self-care behaviors at baseline and follow-up were compared using one-way ANOVA. To examine changes in these outcomes over time within and between groups, Repeated Measures ANOVA was conducted. A significance level of *p* < 0.05 was considered statistically significant.

## Results

A total of 150 patients with type 2 diabetes and limited health literacy were initially enrolled in the study. During follow-up, 3 participants from the SCT + AI photo-novel group (TBG + AIPN), 5 participants from the SCT-based group (TBG), and 4 participants from the control group withdrew and did not continue in the study. Consequently, 138 participants completed the intervention and were included in the final analysis: 47 participants in the SCT + AI photo-novel group (TBG + AIPN), 45 participants in the SCT-based group (TBG), and 46 participants in the control group. The participant flow throughout the study, including enrollment, allocation, follow-up, and analysis, is presented in Fig. 1(CONSORT flow diagram). The study was conducted from April to June 2024.

At baseline, there were no statistically significant differences among the three study groups in terms of demographic and clinical characteristics. The distribution of marital status, gender, education level, economic status, employment, insurance coverage, comorbidities, and family history of diabetes was comparable across the TBG, TBG + AIPN, and the control group (Table 2). The mean age of participants was 50.71 ± 9.36 years in the TBG + AIPN group, 51.38 ± 9.07 years in the control group, and 49.30 ± 12.10 years in the TBG group, with no significant difference (*p* = 0.611). Similarly, there were no significant differences between groups regarding diabetes duration or number of family members, indicating baseline homogeneity among participants.

**Table 2:** Comparison of baseline demographic characteristics across study groups

Variable	Control group	TBG		TBG + AIPN		p-value
	**(Percentage) Frequency**	**(Percentage) Frequency**			**(Percentage) Frequency**	
**Marital status**						0.571
Married	34 (73.9%)		35 (77.8%)		32 (68.1%)	
Single	12 (26.1%)		10 (22.2%)		15 (31.9%)	
**Gender**						0.430
Woman	30 (65.2%)		30 (66.7%)		36 (76.6%)	
Man	16 (34.8%)		15 (33.3%)		11 (23.4%)	
**Education**						0.278
Elementary	11 (23.9%)		11 (24.4%)		16 (34.0%)	
Secondary	20 (43.5%)		26 (57.8%)		18 (38.3%)	
University	15 (32.6%)		8 (17.8%)		13 (27.7%)	
**Economic situation**						0.528
Good	11 (23.9%)		5 (11.1%)		7 (14.9%)	
Average	27 (58.7%)		31 (68.9%)		29 (61.7%)	
Poor	8 (17.4%)		9 (20.0%)		11 (23.4%)	
**Job**						0.635
Unemployed	20 (43.5%)		22 (48.9%)		21 (44.7%)	
Free	1 (2.2%)		1 (2.2%)		3 (6.4%)	
Employee	11 (23.9%)		5 (11.1%)		9 (19.1%)	
**Supplementary insurance**						0.642
yes	23 (50.0%)		24 (53.3%)		28 (59.6%)	
no	23 (50.0%)		21 (46.7%)		19 (40.4%)	
**Other disease**						0.599
yes	23 (50.0%)		22 (48.9%)		19 (40.4%)	
no	23 (50.0%)		23 (51.1%)		28 (59.6%)	
**Family history**						0.601
yes	21 (45.7%)		20 (44.4%)		17 (36.2%)	
no	25 (54.3%)		25 (55.6%)		30 (63.8%)	

### Psychosocial constructs

Analysis of psychosocial constructs revealed significant Group × Time interactions for all measured variables (*p* < 0.001). Post-hoc pairwise comparisons with Bonferroni correction demonstrated distinct patterns of improvement across the three groups. For diabetes knowledge, the TBG + AIPN group showed significantly greater improvement than the TBG group (*p* = 0.040). Both intervention groups demonstrated substantially greater gains in knowledge compared to the control group (*p* < 0.001 for both comparisons). A similar pattern emerged for self-efficacy, where the TBG + AIPN group outperformed the TBG group (*p* = 0.020), and both intervention groups showed significantly better outcomes than the control group (*p* < 0.001).

In terms of outcome expectations, the integrated approach (TBG + AIPN) proved superior to theory-based education alone (*p* = 0.030), while both interventions were significantly more effective than the control condition (*p* < 0.001). Self-regulation outcomes mirrored this trend, with the TBG + AIPN group achieving better results than the TBG group (*p* = 0.020), and both intervention groups showing significant advantages over the control group (*p* < 0.001). Interestingly, for perceived social support, while no significant difference was observed between the two intervention groups (*p* = 0.800), both demonstrated significant improvements compared to the control group (*p* < 0.001).

### Self-care behaviors

The analysis of self-care behaviors revealed a significant Group × Time interaction (*p* < 0.001). Post-hoc tests indicated that the TBG + AIPN group achieved significantly greater improvements in self-care behaviors compared to both the TBG group (*p* = 0.002) and the control group (*p* < 0.001). The TBG group also showed significantly better outcomes than the control group (*p* < 0.001).

These results suggest that the integration of health literacy strategies, particularly the AI-designed visual novel, with theory-based education creates a synergistic effect that significantly enhances the intervention’s impact on both psychosocial constructs and self-care behaviors in patients with type 2 diabetes. Detailed comparisons of the mean scores of study variables between groups and over time, based on repeated-measures ANOVA and post-hoc pairwise tests, are summarized in Table 3.

**Table 3:** Comparison of mean scores of study variables among the three groups over time

Variable	time	TBG+AIPN	TBG	Control group	*p*-value**
		M ± SD	M ± SD	M ± SD	
knowledge	Before training	5.1 ± 1.40	4.9 ± 1.46	5.4 ± 1.97	<0.001
	One month after training	8.6 ± 0.75	7.7 ± 1.17	5.3 ± 1.88	
	Three months after training	8.8 ± 0.75	7.9 ± 1.19	5.5 ± 1.84	
	P-value*	< 0.001	< 0.001	0.162	
Self-efficacy	Before training	62.34 ± 6.93	61.93 ± 5.40	62.34 ± 6.51	< 0.001
	One month after training	76.76 ± 4.43	72.68 ± 4.03	62.28 ± 6.02	
	Three months after training	80.72 ± 5.42	77.31 ± 3.96	62.95 ± 6.01	
	P-value*	< 0.001	< 0.001	0.124	
Outcome Expectations	Before training	25.44 ± 2.06	25.64 ± 1.86	25.8 ± 2.81	< 0.001
	One month after training	29.02 ± 1.53	27.8 ± 1.23	25.17 ± 3.34	
	Three months after training	31.21 ± 2.74	29.1 ± 2.15	25.7 ± 3.29	
	P-value*	< 0.001	< 0.001	0.152	
Self-regulation	Before training	34.3 ± 2.76	33.5 ± 2.87	33.5 ± 5.53	< 0.001
	One month after training	49.1 ± 3.69	46.8 ± 3.47	34.13 ± 6.24	
	Three months after training	52.4 ± 4.11	48.8 ± 3.87	34.5 ± 6.57	
	P-value*	< 0.001	< 0.001	0.117	
Social support	Before training	32.40 ± 5.12	32.97 ± 4.01	32.80 ± 5.81	< 0.001
	One month after training	43.65 ± 8.20	43.44 ± 2.26	31.71 ± 5.04	
	Three months after training	45.87 ± 3.84	43.97 ± 2.77	31.28 ± 4.98	
	P-value*	< 0.001	< 0.001	0.03	
Self-care	Before training	34.58 ± 4.21	34.33 ± 3.70	35.06 ± 6.23	< 0.001
	One month after training	52.50 ± 4.18	48.48 ± 3.89	34.50 ± 5.56	
	Three months after training	56.69 ± 6.32	51.95 ± 3.75	35.69 ± 5.09	
	P-value*	< 0.001	< 0.001	0.120	

* Within-group comparisons of changes in mean scores over time were conducted using repeated-measures ANOVA. ** The p-value in the last column represents the Group × Time interaction obtained from the repeated-measures ANOVA, indicating whether the pattern of change across the three time points differs among the study groups.

### Adverse events

No adverse events or unintended effects were observed in any of the study groups during the trial.

## Discussion

This study examined the impact of educational interventions grounded in Social Cognitive Theory (SCT), supplemented with health literacy strategies, on enhancing self-care behaviors among patients with type 2 diabetes and limited health literacy. Findings indicate that TBG effectively improves knowledge, self-efficacy, self-regulation, and social support. Moreover, integrating health literacy strategies with SCT education significantly amplified these effects, leading to greater improvements in self-care behaviors within this population.

This study demonstrated a significant improvement in knowledge scores among intervention groups following the educational program, with no significant change in the control group. These findings are consistent with previous research highlighting the effectiveness of Social Cognitive Theory (SCT)-based interventions [13] and the role of health literacy strategies such as simplified language and visual aids in enhancing patients’ understanding [35–40]. The integration of SCT with health literacy principles in the present intervention appears to offer a novel and effective approach for improving knowledge among patients with limited health literacy, particularly in the context of diabetes self-care.

This study demonstrated that both the TBG and the TBG+AIPN showed significant improvements in self-efficacy among diabetic patients, with the combined intervention yielding a notably greater increase. These findings suggest that tailoring educational content to participants’ health literacy levels enhances the effectiveness of theory-driven interventions. Consistent with prior research, studies by Ghoreishi et al. and Kaveh et al. [41, 42] have reported positive effects of Social Cognitive Theory-based interventions on self-efficacy in diabetic populations. Furthermore, investigations by Wichit et al. [43], Cavanaugh et al. [44], and Larki [35] emphasize that the use of simplified language, visual aids, and health literacy strategies significantly improves self-efficacy and promotes better self-care behaviors. The greater effectiveness of the combined intervention can be explained through the SCT principle of modeling: storytelling and photo-novels facilitate emotional engagement, identification with role models, and enhanced belief in personal capability, which are critical drivers of self-efficacy.

Outcome expectations significantly influence adherence to self-care behaviors in chronic disease patients [32]. In this study, both intervention groups showed significant increases in outcome expectations one and three months post-intervention, while the control group did not. The TBG+AIPN demonstrated greater improvement than the TBG. These findings were consistent with the studies of Mehdizadeh et al. [40], and Wichit [39], who reported an increase in outcome expectations after the educational intervention. Improving patients’ belief in positive and achievable outcomes is crucial for initiating and maintaining health behaviors. The superior effect observed in the TBG+AIPN highlights the importance of tailoring interventions to patients’ literacy levels using simplified language, visual narratives, and relatable storytelling. For health systems, this suggests that incorporating literacy-sensitive strategies into theory-based programs can more effectively empower patients and improve chronic disease management outcomes.

Self-regulation plays a critical role in sustaining self-care among patients with type 2 diabetes. In this study, both intervention groups showed significant improvements post-intervention, with greater gains observed in the group receiving literacy-sensitive enhancements. These findings align with previous studies (e.g., Kaveh et al. [41], Ghoreishi et al. [42], Wichit et al. [43], Peyman et al. [45] emphasized the positive impact of Social Cognitive Theory-based education and goal-setting strategies on enhancing self-regulatory skills. Importantly, the use of literacy-sensitive strategies such as simple language and photo-novel storytelling appears to have facilitated goal identification, action planning, and self-monitoring among patients with limited health literacy. This supports Bandura’s principle that self-regulation can be strengthened through incremental successes and accessible communication. From a public health perspective, these results underscore the importance of integrating direct self-regulation skill-building into patient education programs, particularly when serving populations with limited literacy. Tailoring content to cognitive capacity through visual tools and simplified formats can enhance internal motivation and long-term behavior maintenance in chronic disease management.

In the present study, following the intervention, both intervention groups (with and without health literacy strategies) showed a significant increase in perceived social support, while a significant decrease was observed in the control group. No statistically significant difference was found between the two intervention groups regarding the effectiveness of the interventions on perceived social support. This may indicate the influence of the group-based educational structure, experience sharing, and participant interaction, regardless of the use of health literacy strategies. Additionally, the role of the educator as an indirect source of psychological support should not be overlooked. Although the use of tools such as photo-novels can be highly effective in enhancing constructs like knowledge or self-efficacy, they may not be sufficient on their own to improve perceived social support, which is more deeply rooted in human relationships and cultural contexts. Previous studies have also confirmed the effectiveness of Social Cognitive Theory-based education in enhancing perceived social support [41, 42, 46]. However, to further improve this construct among patients with limited health literacy, it is recommended that educational interventions be designed with active involvement of family members, peer support groups, and community health facilitators. Moreover, leveraging communication technologies such as virtual peer support groups and messaging platforms can serve as an effective approach, especially in communities with limited access to direct support resources. Overall, these findings highlight the importance of integrating theory-based education with participatory structures. The health system can promote perceived social support by developing family-centered educational programs, strengthening community-based support groups, and utilizing digital platforms to facilitate patient communication. This is particularly crucial in managing chronic conditions among individuals with limited health literacy, as it plays a key role in improving behavioral outcomes and quality of life.

This study aimed to enhance self-care behaviors in patients with type 2 diabetes and limited health literacy by using Social Cognitive Theory (SCT) constructs alongside health literacy strategies. While no baseline differences were observed between groups, post-intervention results showed significant improvements in both intervention groups, with the TBG+AIPN showing greater gains than the TBG. Findings from this study, along with previous evidence [47–49], indicate that educational interventions based on Social Cognitive Theory especially when combined with health literacy strategies such as using simple, understandable language and visual storytelling have a greater impact on improving self-care behaviors in patients with type 2 diabetes who have limited health literacy. These results underscore the importance of aligning educational methods with patients’ health literacy levels, suggesting that relying solely on theoretical models without considering patients’ ability to comprehend and process information may limit the effectiveness of interventions. In the health system, these findings can inform the design of educational programs that are culturally appropriate and tailored to the health literacy levels of their audiences. Programs that incorporate visual, narrative, and participatory approaches can foster more sustainable self-care behaviors, reduce complications and healthcare costs, and improve patients’ quality of life. Therefore, integrating health literacy strategies into patient education policies particularly for vulnerable groups can be an effective step toward health equity and enhancing the impact of care interventions.

An important practical aspect of this study is the AI-designed visual novel, which served as an engaging educational tool to reinforce SCT-based concepts while being accessible for patients with limited health literacy. This novel uses simplified language and culturally relevant characters to facilitate comprehension and behavior change. Although the current manuscript does not provide direct access to these materials, they can be made available upon request for clinical or educational use. Future dissemination efforts should include sharing the AI-generated visual content through online platforms or institutional repositories, enabling other clinicians, educators, and researchers to implement similar interventions in practice. Incorporating such tools into patient education programs may enhance engagement, understanding, and adherence, particularly among populations with low literacy.

The integration of AI-designed educational tools, such as the photonovel developed in this study, provided unique added value beyond traditional storytelling and facilitator-led education. While both intervention groups received identical educational content based on Social Cognitive Theory, the AI-enhanced tool increased engagement and comprehension among participants with limited health literacy by using culturally relevant characters, simplified text, and emotionally expressive visuals. These features allowed participants to better identify with the scenarios and internalize behavioral messages, facilitating deeper learning and motivation to apply self-care practices. Moreover, the interactive use of the photonovel during sessions promoted active discussion and reflection, further reinforcing self-efficacy and self-regulation. Thus, the AI-based photonovel served not only as an instructional medium but also as an adaptive communication strategy, bridging the gap between complex medical concepts and the health literacy level of patients.

Although this study provides important insights, several limitations should be considered when critically interpreting the results. One limitation is the use of the self-reported SDSCA questionnaire to assess diabetes self-care behaviors, which may be subject to response bias and may not fully reflect participants’ actual behaviors. In addition, the study sample was limited to a single city, restricting the generalizability of the findings to other geographic or cultural contexts. Furthermore, the study lacked long-term follow-up, so the sustainability of the intervention effects remains unclear. Future research should consider using more objective measurement tools, include diverse populations from multiple regions, and incorporate long-term follow-up to enhance the reliability and applicability of the findings.

Ultimately, it should be acknowledged that integrating behavioral theories with health literacy strategies presents an effective, flexible, and generalizable approach to enhancing self-care among patients with limited health literacy. This integration can contribute to reducing complications and the burden of chronic diseases within the healthcare system. It is recommended that future research conduct multicenter studies with diverse samples and long-term follow-ups to better assess the sustainability of intervention effects and provide more generalizable results. Such an educational approach may play a critical role in improving healthcare service quality and promoting equitable access to health education.

## Data Availability

The datasets used during the current study are available from the corresponding author on reasonable request.

## Abbreviations

T2DM: Type 2 diabetes mellitus
RCT: Randomized Controlled Trial
SCT: Social Cognitive Theory
TBG: Theory-based group
AIPN: AI-designed photo-novel
S-TOFHLA: Short Test of Functional Health Literacy in Adults
SDSCA: Summary of Diabetes Self-Care Activities
DMSES: The Diabetes Management Self-Efficacy Scale

## References

[1] International diabetes federation (IDF). About diabetes (Facts & figures) 2025. Available from: https://idf.org/about-diabetes/diabetes-facts-figures/.

[2] International diabetes federation (IDF). Iran (Key information) 2024. Available from: https://idf.org/our-network/regions-and-members/middle-east-and-north-africa/members/iran/.

[3] Cheng Z, Xiao Q, Xu Y, Tan L, Qu W, Shen W, et al. Effectiveness of patient-centred care in self-management of type 2 diabetes: a systematic review and meta-analysis. BMC Health Serv Res. 2025;25(1):1–11. 40296007 10.1186/s12913-025-12539-6 PMC12036161

[4] Ali MK, Pearson-Stuttard J, Selvin E, Gregg EW. Interpreting global trends in type 2 diabetes complications and mortality. Diabetologia. 2022;65(1):3–13. 34837505 10.1007/s00125-021-05585-2 PMC8660730

[5] Hite AH. Diabetes: Is There a cure? Health. 2025.

[6] Persson V, Lovén Wickman U, editors. Artificial Intelligence as a Tool for Self-Care in Patients with Type 1 and Type 2 Diabetes—An Integrative Literature Review. Healthcare: MDPI; 2025. 10.3390/healthcare13080950 PMC12026472 40281899

[7] Omar SM, Musa IR, ElSouli A, Adam I. Prevalence, risk factors, and glycaemic control of type 2 diabetes mellitus in eastern Sudan: a community-based study. Ther Adv Endocrinol Metab. 2019;10:2042018819860071. 31275546 10.1177/2042018819860071 PMC6598316

[8] Baum A, Wisnivesky J, Basu S, Siu AL, Schwartz MD. Association of Geographic Differences in Prevalence of Uncontrolled Chronic Conditions With Changes in Individuals’ Likelihood of Uncontrolled Chronic Conditions. JAMA. 2020;324(14):1429–38. 33048153 10.1001/jama.2020.14381 PMC8094427

[9] Dehvan F, Qasim Nasif F, Dalvand S, Ausili D, Hasanpour Dehkordi A, Ghanei Gheshlagh R. Self-care in Iranian patients with diabetes: A systematic review and meta-analysis. Prim Care Diabetes. 2021;15(1):80–7. 32921619 10.1016/j.pcd.2020.08.013

[10] Gerber BS, Brodsky IG, Lawless KA, Smolin LI, Arozullah AM, Smith EV, et al. Implementation and evaluation of a low-literacy diabetes education computer multimedia application. Diabetes Care. 2005;28(7):1574–80. 15983303 10.2337/diacare.28.7.1574

[11] Jerant A, Hanson B, Kravitz RL, Tancredi DJ, Hanes E, Grewal S, et al. Detecting the effects of physician training in self-care interviewing skills: Coding of standardized patient (SP) visit recordings versus SP post-visit ratings. Patient Educ Couns. 2017;100(2):367–71. 27578271 10.1016/j.pec.2016.08.021 PMC5318274

[12] Šorgo AŠAŠaA. The worldview of preservice and in-service teachers about health education. J Elementary Educ. 2020;13(2).

[13] Ghoreishi M-S, Vahedian-Shahroodi M, Esmaily H, Tehrani H. Predictive Factors Related To Self-Care Behaviors among Type2 Diabetic Patients by Using Social Cognitive Model. Iran J Health Educ Health Promotion. 2018;6(3):241–50.

[14] Hassani L, Yari A, Mohseni S. The Effect of Educational Intervention Based on Social Cognitive Theory on Self-care Behavior of Patients with Diabetes Referring to Comprehensive Health Service Centers in Fasa. Iran J Health Educ Health Promotion. 2025;13(2):173–88.

[15] Alidosti M, Tavassoli E. Role of self-efficacy, outcome expectation, and outcome expectancy in promoting oral health behaviors in adolescent girls. J Educ Health Promot. 2020;9:254. 33224998 10.4103/jehp.jehp_784_19 PMC7657404

[16] shabibi. mansourian m, abedzadeh ms, sayehmiri k. The Status of Self-Care Behaviors in Patients with Type 2 Diabetes in the City of Ilam in 2014. J Ilam Univ Med Sci. 2016;24(2):63–71.

[17] Fleary SA, Joseph P, Pappagianopoulos JE. Adolescent health literacy and health behaviors: A systematic review. J Adolesc. 2018;62:116–27. 29179126 10.1016/j.adolescence.2017.11.010

[18] Fransen MP, Beune EJ, Baim-Lance AM, Bruessing RC, Essink‐Bot ML. Diabetes Self‐Management support for patients with low health literacy: perceptions of patients and providers. J diabetes. 2015;7(3):418–25. 25042519 10.1111/1753-0407.12191

[19] Nutbeam D. Health literacy as a public health goal: a challenge for contemporary health education and communication strategies into the 21st century. Health Promot Int. 2000;15(3):259–67.

[20] Kountz DS. Strategies for improving low health literacy. Postgrad Med. 2009;121(5):171–7. 19820287 10.3810/pgm.2009.09.2065

[21] Houts PS, Doak CC, Doak LG, Loscalzo MJ. The role of pictures in improving health communication: a review of research on attention, comprehension, recall, and adherence. Patient Educ Couns. 2006;61(2):173–90. 16122896 10.1016/j.pec.2005.05.004

[22] Adegboye M. Impact of artificial intelligence on health information literacy: guidance for healthcare professionals. Libr Hi Tech News. 2024;41(7):1–5.

[23] Alowais SA, Huang I-W. Revolutionizing Healthcare: The Role of Artificial Intelligence in Clinical Practice. Angle Health Law Rev. 2024;94:95–132.

[24] Baker DW, Williams MV, Parker RM, Gazmararian JA, Nurss J. Development of a brief test to measure functional health literacy. Patient Educ Couns. 1999;38(1):33–42. 14528569 10.1016/s0738-3991(98)00116-5

[25] Han H-R, Kim J, Kim MT, Kim KB. Measuring health literacy among immigrants with a phonetic primary language: a case of Korean American women. J Immigr Minor Health. 2011;13(2):253–9. 20585985 10.1007/s10903-010-9366-0 PMC3010254

[26] Connor M, Mantwill S, Schulz PJ. Functional health literacy in Switzerland—Validation of a German, Italian, and French health literacy test. Patient Educ Couns. 2013;90(1):12–7. 23089240 10.1016/j.pec.2012.08.018

[27] Rahimi B, Tavassoli E. Measuring Health Literacy of Elementary School Teachers in Shahrekord. J Health Lit. 2019;4(1):25–32.

[28] Toobert DJ, Hampson SE, Glasgow RE. The summary of diabetes self-care activities measure: results from 7 studies and a revised scale. Diabetes Care. 2000;23(7):943–50. 10895844 10.2337/diacare.23.7.943

[29] Wu SF, Courtney M, Edwards H, McDowell J, Shortridge-Baggett LM, Chang PJ. Self-efficacy, outcome expectations and self-care behaviour in people with type 2 diabetes in Taiwan. J Clin Nurs. 2007;16(11c):250–7. 17931318 10.1111/j.1365-2702.2006.01930.x

[30] Bijl JV, Poelgeest-Eeltink AV, Shortridge-Baggett L. The psychometric properties of the diabetes management self-efficacy scale for patients with type 2 diabetes mellitus. J Adv Nurs. 1999;30(2):352–9. 10457237 10.1046/j.1365-2648.1999.01077.x

[31] Azita N, Rahim T. The diabetes management self-efficacy scale: translation and psychometric evaluation of the iranian version. Nurs Pract Today. 2015;1(1).

[32] Reisi M, Fazeli H, Mahmoodi M, Javadzadeh H. Application of the Social Cognitive Theory to Predict Self-Care Behavior among Type 2 Diabetes Patients with Limited Health Literacy. J Health Lit. 2021;6(2):21–32.

[33] Eigenmann C, Skinner T, Colagiuri R. Development and validation of a diabetes knowledge questionnaire. Practical Diabetes Int. 2011;28(4):166–d70.

[34] Talbot F, Nouwen A, Gingras J, Gosselin M, Audet J. The assessment of diabetes-related cognitive and social factors: the Multidimensional Diabetes Questionnaire. J Behav Med. 1997;20(3):291–312. 9212382 10.1023/a:1025508928696

[35] Larki A. Effects of educational intervention strategies based on self-care health literacy hypertensive patients with limited health literacy: An Application of Health Belief Model. Bushehr: Bushehr University of Medical Sciences; 2017.

[36] Mirzaei A, Ramezankhani A, Taheri Tanjani P, Ghaffari M, Jorvand R, Bazyar M, et al. The effectiveness of health literacy based educational intervention on nutritional outcomes of elderly. Salmand: Iran J Ageing. 2020;15(3):324 – 37.

[37] Michielutte R, Bahnson J, Dignan MB, Schroeder EM. The use of illustrations and narrative text style to improve readability of a health education brochure. J Cancer Educ. 1992;7(3):251–60. 1419592 10.1080/08858199209528176

[38] Yin HS, Gupta RS, Mendelsohn AL, Dreyer B, van Schaick L, Brown CR, et al. Use of a low-literacy written action plan to improve parent understanding of pediatric asthma management: A randomized controlled study. J Asthma. 2017;54(9):919–29. 28045551 10.1080/02770903.2016.1277542

[39] Stableford S, Mettger W. Plain language: a strategic response to the health literacy challenge. J Public Health Policy. 2007;28(1):71–93. 17363939 10.1057/palgrave.jphp.3200102

[40] Grene M, Cleary Y, Marcus-Quinn A. Use of Plain-Language Guidelines to Promote Health Literacy. IEEE Trans Prof Commun. 2017;60:384–400.

[41] Kaveh MH, Montazer M, Karimi M, Hassanzadeh J. Effects of a theory-based training program with follow-up home visits on self-management behavior, glycemic index, and quality of life among Iranian patients with type 2 diabetes mellitus. BMC Public Health. 2022;22(1):1559. 35974352 10.1186/s12889-022-13959-3 PMC9379227

[42] Ghoreishi MS, Vahedian-Shahroodi M, Jafari A, Tehranid H. Self-care behaviors in patients with type 2 diabetes: Education intervention base on social cognitive theory. Diabetes Metab Syndr. 2019;13(3):2049–56. 31235135 10.1016/j.dsx.2019.04.045

[43] Wichit N, Mnatzaganian G, Courtney M, Schulz P, Johnson M. Randomized controlled trial of a family-oriented self-management program to improve self-efficacy, glycemic control and quality of life among Thai individuals with Type 2 diabetes. Diabetes Res Clin Pract. 2017;123:37–48. 27918976 10.1016/j.diabres.2016.11.013

[44] Cavanaugh K, Wallston KA, Gebretsadik T, Shintani A, Huizinga MM, Davis D, et al. Addressing literacy and numeracy to improve diabetes care: two randomized controlled trials. Diabetes Care. 2009;32(12):2149–55. 19741187 10.2337/dc09-0563 PMC2782967

[45] Peyman N, Taghipour A, Mahdizadeh M, Esmaeely H. The effect of educational intervention based on self-regulation strategies on physical activity in women with type 2 diabetes. Evid Based Care. 2013;2(4):7–17.

[46] Amini Moridani M, Tol A, Sadeghi R, Mohebbi B, Azam K. Assessing the Effect of Family-based Intervention Education Program on Perceived Social Support among Older Adults with Type 2 Diabetes: Application of Social Cognitive Theory. J Nurs Educ. 2015;2(3):30–40.

[47] Negarandeh R, Mahmoodi H, Noktehdan H, Heshmat R, Shakibazadeh E. Teach back and pictorial image educational strategies on knowledge about diabetes and medication/dietary adherence among low health literate patients with type 2 diabetes. Prim Care Diabetes. 2013;7(2):111–8. 23195913 10.1016/j.pcd.2012.11.001

[48] Ounnapiruk L, Wirojratana V, Meehatchai N, Turale S. Effectiveness of a behavior modification program for older people with uncontrolled type 2 diabetes. Nurs Health Sci. 2014;16(2):216–23. 23991917 10.1111/nhs.12089

[49] Sze WT, Waki K, Enomoto S, Nagata Y, Nangaku M, Yamauchi T, et al. StepAdd: A personalized mHealth intervention based on social cognitive theory to increase physical activity among type 2 diabetes patients. J Biomed Inf. 2023;145:104481. 10.1016/j.jbi.2023.104481 37648101

